# Impact of Positive Airway Pressure on International Restless Legs Syndrome Score in Sleep Disordered Breathing

**DOI:** 10.3390/jcm8122212

**Published:** 2019-12-14

**Authors:** Seetha Lakshmanan, Nicolas R. Thompson, Maeve Pascoe, Reena Mehra, Nancy Foldvary-Schaefer, Irene L. Katzan, Harneet K. Walia

**Affiliations:** 1 Sleep Disorders Center, Neurological Institute, Cleveland Clinic, Cleveland, OH 44195, USA; seetha.hkl@gmail.com (S.L.); pascoem2@ccf.org (M.P.); mehrar@ccf.org (R.M.); foldvan@ccf.org (N.F.-S.); 2 Department of Qualitative Health Sciences, Cleveland Clinic, Cleveland, OH 44195, USA; thompsn@ccf.org; 3 Neurological Institute Center of Research Outcomes, Cleveland Clinic, Cleveland, OH 44195, USA; katzani@ccf.org

**Keywords:** restless legs syndrome (RLS), sleep disordered breathing (SDB), positive airway pressure (PAP), International Restless Legs Syndrome score (IRLS)

## Abstract

Study Objective: Studies have shown increased prevalence of restless legs syndrome (RLS) in sleep disordered breathing (SDB), however limited data have focused on the impact of SDB therapy on RLS. We hypothesize that positive airway pressure (PAP) will improve the International Restless Legs Syndrome (IRLS) score among SDB patients compared to patients without PAP. Methods: Patients with AHI ≥ 5 who responded positively to a RLS qualifier question from January 2010 to May 2015 were included in this retrospective study. IRLS score was used to measure RLS symptom severity. Two-sample *t*-tests and one-way analysis of variance were used to compare changes in IRLS score and linear regression models were created to examine IRLS change with PAP use and PAP adherence (PAP usage ≥4 h nightly for ≥70% of nights), adjusting for potential confounders. Results: In 434 patients (51.9 ± 13.4years, 50.5% female, 77.6% Caucasian; 325 PAP, 109 control), IRLS scores improved from baseline to follow-up, with the PAP group achieving significant improvement after adjustment for covariates (difference in IRLS: −1.8 (CI −3.6,0.00), *p* = 0.050). In self-reported PAP adherent patients, IRLS improvement was greater than controls (−5.3 ± 7.4 vs. −2.7 ± 7.6 respectively, *p* = 0.045), and comparable to non-adherent patients (−5.3 ± 7.4 vs. −3.0 ± 7.0, *p* = 0.091). Conclusions: Among SDB patients with a positive RLS qualifier, those who used PAP therapy achieved significantly greater improvement in IRLS scores than patients who did not use PAP, with more significant changes in the PAP adherent group. This is the first large clinical study to examine these relationships, providing a basis for future prospective interventional trials and informing clinicians of expected improvement in IRLS score in PAP treated SDB populations.

## 1. Introduction

Restless legs syndrome (RLS), also known as Willis–Ekbom disease, is a chronic sensorimotor disorder with a prevalence of approximately 10% in the general population, yet often underdiagnosed and undertreated [1]. RLS has adverse downstream consequences, including impairment in quality of life, trouble falling asleep, daytime sleepiness, and difficulty in concentration and learning [2].

The association between RLS and sleep disordered breathing (SDB) has recently drawn increasing attention [3], but the link between the two disorders remains unclear. It is suggested that RLS is more prevalent in SDB patients than in the general population; the Icelandic Sleep Apnea Cohort reported RLS in 23–36% of SDB patients, compared to 13–25% of controls [4]. However, another prospective study found RLS less prevalent in SDB patients than the general population, observing newly diagnosed RLS in 1 out of every 12 patients undergoing polysomnography (PSG) for sleep apnea [5]. Regardless, the recognition of RLS among SDB patients is clinically important, as SDB may worsen RLS symptoms [5].

Several studies have examined the impact of SDB treatment on RLS [3,6], however existing studies had small sample sizes and lacked control groups, thereby limiting generalizability. Given the clinical importance of identifying RLS in SDB patients and the potential treatment effects of SDB on RLS severity, we aimed to evaluate RLS severity using the International Restless Legs Syndrome (IRLS) score among SDB patients using positive airway pressure (PAP) therapy and controls in a large clinical population. We hypothesize that PAP therapy would improve IRLS scores in SDB patients regardless of severity, and that greater improvement would be seen in those adherent to PAP therapy.

## 2. Methods

### 2.1. Patient Population

Patients who presented to the Cleveland Clinic Sleep Disorders Center for any sleep related disorders, from 1 January 2010 to 31 May 2015 were included if they met the following criteria: (1) ≥18 years; (2) available polysomnography (PSG) data, with apnea hypopnea index (AHI) of ≥5 confirming sleep apnea; (3) completed patient-reported outcomes (PRO) data 0–90 days before PSG (baseline) and 30–365 days after PSG (follow-up) including an affirmative response to the RLS qualifier question and the IRLS; and (4) no PAP therapy prior to baseline. Patients on PAP therapy at follow-up were included in the PAP study group; those not using PAP, regardless of other SDB therapies, were included in the control group. The study was approved by the Institutional Review Board of the Cleveland Clinic.

### 2.2. Data Collection from the Electronic Health Record

Baseline demographic, comorbidity, and PSG data were obtained through the electronic health record (EHR; Epic Systems Corporation^®^, Verona, Wisconsin). This included the patient’s age (years), sex, body mass index (BMI; kg/m^2^), AHI, neck circumference (cm), race (Caucasian, African American, other), socioeconomic status, smoking status (current, former, never), caffeine consumption (yes/no), RLS medication use (yes/no), exacerbating medication use (yes/no), and history of comorbid conditions. Socioeconomic status was measured using median income by zip code using 2010 United States Census data. RLS medications included iron supplements, dopaminergic agents, alpha-2-delta ligands, opioids, benzodiazepines, and magnesium supplements. Exacerbating medications included antidepressants, antipsychotics, anti-emetics, centrally acting histamines, and caffeine. Medications were obtained by chart review at baseline and follow-up.

PSG data was acquired using the Polysmith (©Nihon Kohden Corporation, Tokyo, Japan) system following standard clinical guidelines. Nasal airflow and nasal pressure were measured using oronasal thermistor and nasal cannula, respectively. Hypopnea was defined as a reduction in airflow ≥50% in the nasal pressure channel for ≥10 s resulting in an arousal or ≥3% oxygen desaturation and apnea was defined as a decrease in amplitude of oronasal thermistor signal by 90% for ≥10 s, based on American Academy of Sleep Medicine (AASM) event definition criteria [7].

### 2.3. Data Collection from the Cleveland Clinic Knowledge Program

The RLS qualifier, IRLS score, and PAP adherence data were obtained from the Cleveland Clinic Knowledge Program database. The Knowledge Program© (KP) is an electronic platform for systematic collection of disease-based patient reported outcomes via administration on tablet computers or through the EHR patient portal (MyChart, Epic Systems^®^) [8]. The RLS qualifier asked: “Do you ever have an unpleasant, restless feeling in your legs that can be relieved by walking or movement, particularly at night?” [9]. This question had high sensitivity and specificity for RLS in a prior validation study [10].

After RLS qualification and study entry, the IRLS questionnaire was given to patients during their clinic visit to assess RLS symptom severity by way of questions that target RLS diagnosis criteria: (i) strong urge to move the legs in association with paresthesia and disagreeable sensations, such as crawling, aching, or burning in the legs; (ii) associated sensations are relieved by movement; (iii) symptoms have circadian rhythmicity and are worse at night; and (iv) symptoms are worse during periods of rest, especially long periods of inactivity [11]. This well-validated [12], 10-item survey asked patients to rate their symptoms on a Likert-type scale from 0–4. A total score of 0 indicates no RLS, 1–10, mild; 11–20, moderate; 21–30, severe; and 30–40, very severe RLS [13]. While clinical improvements can be seen with less change [12], a decrease of 4.2–6 points is indicative of clinically significant improvement [14,15].

Self-reported PAP adherence was determined from patient responses to questions on PAP usage (yes/no), number of days of PAP usage per week, number of hours of PAP usage per day, and average sleep time (<6 h, 6–9 h, ≥9 h), and objective PAP adherence was determined from data from durable medical equipment companies. Adherence was measured during 30–365 days after PSG (follow-up) and is defined as PAP use ≥4 h on ≥70% of nights, per Centers for Medicare and Medicaid Services (CMS) criteria [16].

### 2.4. Statistical Analyses

Descriptive statistics for the entire cohort and stratified by treatment group (PAP and control) were computed. Two-sample *t*-tests and Mann–Whitney U tests were used to compare continuous variables and Fisher’s exact tests were used to compare categorical variables. Similar tests were performed to compare patients who were included and excluded from the analysis.

Unadjusted means for the baseline, follow-up, and changes in IRLS score were computed for all patients and stratified by treatment group and by PAP adherence (both self-report and objective). Paired *t*-tests were used to determine whether within-group scores significantly improved after PAP treatment. Two-sample *t*-tests and one-way analysis of variance were used to compare whether change in IRLS score differed between PAP and control groups and between PAP-adherent, PAP-non-adherent, and control groups, respectively. Mann–Whitney U test was used to compare PAP adherence to severity of AHI, while Spearman correlation was used to analyze the correlation between severity of RLS and AHI.

Separate linear regression models evaluated the impact of potential confounders on IRLS score change including age, sex, race, smoking status, BMI, baseline AHI, RLS medication, and exacerbating medication use at baseline and follow-up, caffeine consumption, average sleep time, and days from PSG to follow-up. The following comorbidities were also included as separate covariates: coronary artery disease, cancer, chronic renal failure, diabetes, hypertension, and stroke. When making pairwise comparisons between groups, we corrected for multiple testing using the Bonferroni correction.

The distributions of RLS and exacerbating medication use at baseline and at follow-up were examined by creating 2 × 2 tables. When the sum of discordant pairs was ≥25, McNemar’s test was used to examine marginal homogeneity, and when the sum of discordant pairs was <25, exact binomial method was used [17].

To account for missing covariate data, multiple imputation was used to create and analyze 10 imputed datasets. Incomplete variables were imputed under fully conditional specification using the default settings of the Multiple Imputation by Chained Equations (mice) 2.30 package [18,19]. Model parameters were estimated with multivariable linear regression applied to each imputed dataset separately. All computations were done in R, version 3.4.1 (R Core Team, 2017). All tests were two-sided and *p*-values less than 0.05 were considered statistically significant.

## 3. Results

**Figure 1:**
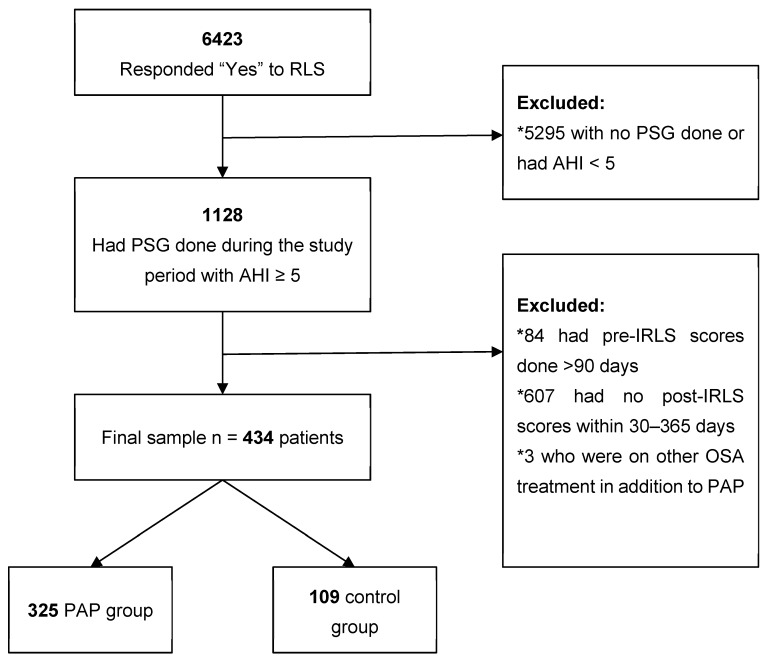
Flow diagram of study sample, starting with 6423 patients that responded “yes” to the RLS qualifier and resulting in a final sample of 434 patients. Of these included patients, 325 were included in the PAP group and 109 in the control group.

From the 6423 patients who responded “Yes” to the RLS qualifier question and completed the IRLS questionnaire at least once during the study period, 434 (age 51.9 ± 13.4 years, 50.5% female, 77.6% Caucasian) met inclusion criteria (Figure 1). Sample characteristics stratified by group (325 PAP, 109 control) are shown in Table 1. Compared to controls, PAP patients were older, were more likely to be male, had higher AHI, and presented for follow-up significantly later. Of the PAP-treated patients, 96.3% had self-reported adherence data at follow-up and 75.7% of those patients reported being adherent. Objective adherence data was available in 130 patients, of which 80 (61.5%) were objectively adherent. Among the 125 patients who had both self-reported and objective adherence data available, agreement between objective and self-reported adherence was 75.2%. Similar tests were performed to compare patients who were included and excluded from the analysis: those included were more likely to be Caucasian and had a higher AHI, with no other significant differences found.

### 3.1. Effect of PAP Treatment on IRLS Score Change

Unadjusted means for baseline, follow-up, as well as the mean change in IRLS scores for both PAP and control groups are presented in Table 2. Within-group IRLS change was significant for both groups (*p <* 0.001), with moderate effect sizes for each. The PAP group achieved clinically significant IRLS improvement (−4.8 ± 7.5) while the control group did not (−2.7 ± 7.6). Both before and after adjustment for covariates, PAP treated patients had significantly more improvement in mean IRLS score than controls (*p* = 0.014 and *p* = 0.050 respectively). The PAP group had a higher average AHI, however severity of AHI and severity of RLS were not correlated in our sample. The Spearman correlation between AHI and baseline IRLS score was 0.021 (*p* = 0.665). Among patients with AHI < 15 (*n =* 175), the average change in IRLS score was −1.43 (*p* = 0.030), while patients with AHI ≥ 15 (*n =* 259) had an average change in IRLS score of −2.06 (*p* < 0.001). Thus, RLS patients with mild sleep apnea syndrome were also responsive to PAP. The mean changes in IRLS score for patients with AHI < 15 vs. AHI ≥ 15 were not significantly different (*p* = 0.459).

### 3.2. Effect of PAP Self-Report Adherence on IRLS Score Change

Covariate-adjusted and unadjusted changes in IRLS score for PAP adherent, non-adherent, and control groups are presented in Table 3. Within-group changes were significant for all groups (*p <* 0.001), but only the PAP adherent group achieved clinically significant improvement (PAP adherent −5.3 ± 7.4 vs. non-adherent−3.0 ± 7.0 vs. −2.7 ± 7.6). After adjustment for covariates and Bonferroni correction, PAP adherent patients had significantly greater IRLS score change than controls (*p* = 0.045). However, even though PAP non-adherent patients and controls did not have significantly different score changes and PAP adherent patients had greater IRLS score improvement numerically, their improvement was not significantly greater than that of PAP non-adherent patients (*p* = 0.091). The median AHI in PAP adherent and non-adherent patients was 22.1 (11.0–49.4) and 21.0 (9.0–40.5), respectively. AHI distributions were not significantly different for adherent and non-adherent patients (*p* = 0.324).

### 3.3. Effect of PAP Objective Adherence on IRLS Score Change

Presented in Table 4 is unadjusted change in IRLS for objectively PAP adherent, non-adherent, and control groups. Within-group changes were again significant for all groups (*p <* 0.001 for all). Estimated changes and between-group differences were fairly similar to those observed in self-reported adherence data, except that the change in IRLS score was larger in the non-adherent group. None of the between-group differences were statistically significant, both before and after adjustment for covariates and after Bonferroni correction for multiple testing.

### 3.4. RLS/Exacerbating Medication Usage

Table 5 shows RLS medication usage and exacerbating medication usage at baseline and follow-up. In the entire cohort, 42.6% used RLS medication at both baseline and follow-up, 40.1% did not use RLS medication at baseline or follow-up, 3.9% used RLS medication at baseline but not at follow-up, and 13.4% did not use RLS medication at baseline but started one before their follow-up visit. The proportion of patients who used RLS medication at follow-up was significantly greater than the proportion that used at baseline (*p <* 0.001), and this relationship was also seen in the PAP group (*p <* 0.001) but not in the control group (*p* = 0.791). For exacerbating medications, the proportion of patients who used medication at follow-up was significantly greater than baseline for the entire cohort (*p* = 0.013), but not for the PAP or control groups individually.

## 4. Discussion

To our knowledge, this is the first large scale clinical study characterizing the response of RLS symptoms to PAP therapy in SDB patients with subjective and objective PAP adherence data compared to a control group. We found that any PAP usage is associated with IRLS score improvement regardless of AHI distributions, even after careful consideration of a range of confounding factors. Moreover, we found that PAP adherent patients achieved greater IRLS score improvement than non-adherent patients and controls; non-adherent patients achieved no greater improvement than controls, and PAP use/adherence was associated with clinically significant IRLS score improvement.

To date, few studies have reported effects of PAP therapy in SDB patients on RLS. One retrospective study involving 17 patients newly diagnosed with SDB and RLS showed improvement in IRLS scores after three months of PAP treatment [6], and another involving 28 patients with obstructive sleep apnea (OSA) and RLS showed significant decrease in RLS symptoms in 71% after PAP therapy for OSA [3]. These studies demonstrated the usefulness of PAP therapy in SDB patients with RLS, but had small sample sizes, did not adjust for potential confounding factors, and did not have a control group comparator. Our study demonstrated similar results in a larger population, adjusted for covariates, included a control group, and stratified analysis based on PAP adherence.

Given that sleep impairments and fragmentation are known triggers for RLS symptoms [20], improved sleep consolidation resulting from PAP therapy and resolution of SDB symptoms may be the cause of the observed improvements in RLS severity. A recent case report highlighted that the use of upper airway stimulation device as an alternative treatment for OSA had also shown significant improvement in both OSA and RLS symptoms [21]. At a more granular level, the relationship between RLS and SDB may be attributable to intermittent hypoxia, as respiratory event-associated hypoxia may cause dysfunction of the dopaminergic pathway [22]. This postulate is supported by the high prevalence (30–40%) of RLS among those with moderate to severe chronic obstructive pulmonary disease (COPD), association of smoking and RLS in epidemiology studies, increased use of inhalative respiratory medications in RLS populations [23], and the clinical experience of worsening RLS with acute exposure to high altitude [24]. Specifically, a 3-fold higher prevalence of RLS has been observed in those with COPD compared to age and sex-matched controls [25]. Transcutaneous oxygen pressure in the legs (and not the trunk) is lower in patients with RLS than healthy controls [26], which provides a potential mechanistic basis for our findings.

Our study is strengthened by the use of a large sample size including a control group, rigorous electronic data collection, and adjustment for potential confounding factors. This study was also the first to utilize standardized IRLS questionnaires collected systematically in a clinic-based setting in accordance with AASM quality measures for RLS [12], and the first to evaluate PAP adherence in relation to IRLS score change. However, our study has limitations. The retrospective design limited our ability to account for and mitigate selection bias and given that RLS classification is based on subjective experience, the use of PAP therapy for any length of time could have improved IRLS scores by way of the placebo effect. Our study did not review oxygen desaturation index data as it was not readily available for analysis. We have thus relied on AHI as a predictor for hypoxia. Proportionally greater RLS medication use was seen at follow-up than baseline in the PAP group but not in controls, even though medication use was adjusted for in our multivariable models. Arguably, one would think the patients who are more adherent with PAP would also naturally be more compliant with RLS medication [27,28]. Although the control group may potentially include patients receiving non-PAP therapy, findings would be expected to be biased toward the null, therefore results may reflect an underestimation of the comparative benefit or PAP treatment on RLS symptoms. We were not able to verify any underlying nasal problems which could hinder PAP adherence. Additionally, less than half of our PAP cohort had objective adherence data available. Although similar trends were observed in the subset of patients with objective adherence data, our study lacked power to observe significant differences.

## 5. Conclusions

This study suggests that PAP therapy improves RLS symptoms in a large, clinical population with SDB. It also highlights the importance of RLS screening in SDB patients and continuous monitoring of RLS symptoms over time. Future prospective intervention-based studies are needed to verify these findings, including identification of optimal strategies to sequentially implement treatment for RLS and SDB. These findings also set the stage for further research to evaluate biomarkers in RLS/SDB patients with and without PAP, as well as experimental studies to elucidate mechanistic underpinnings of these relationships.

## Figures and Tables

**Table 1:** Descriptive statistics for cohort and stratified by treatment group.

	All Patients *N* = 434	PAP *N* = 325	Control *N* = 109	*p*-Value
Age, years	51.9 ± 13.4	53.0 ± 13.0	48.9 ± 14.0	0.008
Female	219 (50.5%)	154 (47.4%)	65 (59.6%)	0.035
Caucasian	337 (77.6%)	258 (79.4%)	79 (72.5%)	0.087
Married	242 (55.8%)	190 (58.5%)	52 (47.7%)	0.170
Median income (×$1000)	53.3 ± 17.5	53.9 ± 17.6	51.6 ± 17.3	0.244
Smoking status				
Current	48 (11.1%)	34 (10.5%)	14 (12.8%)	0.473
Former	142 (32.7%)	111 (34.2%)	31 (28.4%)	
Never	193 (44.5%)	142 (43.7%)	51 (46.8%)	
Missing	51 (11.8%)	38 (11.7%)	13 (11.9%)	
Coronary artery disease	32 (7.4%)	24 (7.4%)	8 (7.3%)	1.000
Cancer	76 (17.5%)	55 (16.9%)	21 (19.3%)	0.563
Chronic renal failure	15 (3.5%)	10 (3.1%)	5 (4.6%)	0.544
Depression	116 (26.7%)	81 (24.9%)	35 (32.1%)	0.169
Diabetes	98 (22.6%)	68 (20.9%)	30 (27.5%)	0.185
Hypertension	168 (38.7%)	124 (38.2%)	44 (40.4%)	0.733
Stroke	15 (3.5%)	8 (2.5%)	7 (6.4%)	0.067
AHI	20 (9,44)	22 (11,49)	13 (8,32)	<0.001
BMI, kg/m^2^	33 (28,38)	33 (29,38)	33 (27,39)	0.389
Neck circumference, cm	40 (37, 43.25)	40 (38, 43.5)	39.5 (36,43)	0.101
Family history of RLS	48 (11.1%)	35 (10.8%)	13 (11.9%)	0.726
RLS medications	202 (46.5%)	146 (44.9%)	56 (51.4%)	0.268
Exacerbating medications	198 (45.6%)	146 (44.9%)	52 (47.7%)	0.657
Any caffeine consumption	325 (74.9%)	252 (77.5%)	73 (67.0%)	0.054
Days from baseline to PSG	23.5 (6,41)	23 (7,38)	25 (5,44)	0.879
Days from PSG to follow-up	113 (75,169)	125 (86,172)	71 (42,131)	<0.001
Baseline IRLS Score	16.2 ± 8.3	16.3 ± 8.3	15.9 ± 8.2	0.596
Baseline average sleep time				
<7 h	286 (65.9%)	216 (66.5%)	70 (64.2%)	0.615
7–9 h	112 (25.8%)	83 (25.5%)	29 (26.6%)	
>9 h	27 (6.2%)	19 (5.8%)	8 (7.3%)	

Statistics presented as Mean ± SD, Median [P25,P75], or N(column%). PAP: positive airway pressure; RLS: restless leg syndrome; SD: standard deviation; IQR: interquartile range; IRLS: International Restless Leg Syndrome Scale; ESS: Epworth Sleepiness Scale; PHQ-9: Patient Health Questionnaire; PSG: polysomnography.

**Table 2:** Change in IRLS score by group.

	PAP *N =* 325	Control *N =* 109	Estimated Difference	*p*-Value	Estimated Difference, Adjusted †	*p*-Value
Baseline	16.3 ± 8.3	15.9 ± 8.2				
Follow-up	11.5 ± 8.9	13.2 ± 8.4				
Change	−4.8 ± 7.5	−2.7 ± 7.6	−2.1 (−3.7, −0.4)	0.014	−1.8 (−3.6, 0.00)	0.050
Effect Size	−0.57	−0.33				

Statistics are presented as Mean ± SD or Value (95% CI). PAP: positive airway pressure, CI: confidence interval. †—Covariates included in multivariable model: age, sex, race, smoking status, body mass index, apnea hypopnea index, RLS medication use, exacerbating medication use, any caffeine consumption, coronary artery disease, cancer, chronic renal failure, diabetes, hypertension, stroke, average sleep time, days from polysomnography to follow-up.

**Table 3:** Change in IRLS score by self-reported PAP adherence.

	PAP Adherent *N =* 237	PAP Non-Adherent *N =* 76	Control *N* = 109	PAP Adherent vs. Control Estimated Difference	PAP Non-Adherent vs. Control Estimated Difference	PAP Adherent vs. PAP Non-Adherent Estimated Difference
Baseline	16.4 ± 8.3	15.4 ± 8.4	15.9 ± 8.2			
Follow-up	11.1 ± 8.3	12.4 ± 9.9	13.2 ± 8.4			
Change, unadjusted	−5.3 ± 7.4	−3.0 ± 7.0	−2.7 ± 7.6	−2.6 (−4.4, −0.7) *p* = 0.010	−0.3 (−2.7, 2.1) *p* > 0.999	−2.3 (−4.4, −0.2) *p* = 0.065
Change, adjusted †				−2.3 (−4.3, −0.3) *p* = 0.045	−0.1 (−2.6, 2.5) *p* > 0.999	−2.2 (−4.9, 0.0) *p* = 0.091
Effect Size	−0.64	−0.35	−0.32			

Statistics are presented as Mean ± SD or value (95% CI). The pairwise group comparison confidence intervals and *p*-values have been corrected for multiple comparisons using the Bonferroni correction. †—Covariates included in multivariable model: age, sex, race, smoking status, body mass index, apnea hypopnea index, RLS medication use, exacerbating medication use, any caffeine consumption, coronary artery disease, cancer, chronic renal failure, diabetes, hypertension, stroke, average sleep time, days from polysomnography to follow-up.

**Table 4:** Change in IRLS score by objective PAP adherence.

	PAP Adherent *N =* 80	PAP Non-Adherent *N =* 50	Control *N =* 109	PAP Adherent vs. Control	PAP Non-Adherent vs. Control	PAP Adherent vs. PAP Non-Adherent
				**Estimated Difference**	**Estimated Difference**	**Estimated Difference**
Baseline	15.4 (7.1)	16.4 (8.7)	15.9 (8.2)			
Follow-up Imp	10.1 (7.5)	12.1 (9.4)	13.2 (8.4)			
Change, unadjusted	−5.2 (7.8)	−4.4 (6.1)	−2.7 (7.6)	−2.5 (−4.8, −0.2) *p* = 0.066	−1.7 (−4.4, 0.9) *p* = 0.481	−0.8 (−3.5, 2.0) *p* > 0.999
Change, adjusted †				−2.0 (−4.6, 0.6) *p* = 0.292	−1.2 (−4.2, 1.7) *p* > 0.999	−0.8 (−3.7, 2.2) *p* > 0.999
Effect Size	−0.73	−0.51	−0.33			

Statistics are presented as Mean ± SD or value (95% CI). The pairwise group comparison confidence intervals and *p*-values have been corrected for multiple comparisons using the Bonferroni correction. †—Covariates included in multivariable model: age, sex, race, smoking status, body mass index, apnea hypopnea index, RLS medication use, exacerbating medication use, any caffeine consumption, coronary artery disease, cancer, chronic renal failure, diabetes, hypertension, stroke, average sleep time, days from polysomnography to follow-up.

**Table 5:** Distributions of RLS medication use at baseline and follow-up for all patients and stratified by treatment group.

			Follow-Up					
			All Patients		PAP Group		Control Group	
			No	Yes	No	Yes	No	Yes
Baseline	RLS Medication Use	No	174 (40.1%)	58 (13.4%)	129 (39.7%)	50 (15.4%)	45 (41.3%)	8 (7.3%)
		Yes	17 (3.9%)	185 (42.6%)	11 (3.4%)	135 (41.5%)	6 (5.5%)	50 (45.9%)
		*p*-Value	<0.001		<0.001		0.791 †	
	Exacerbating Medication Use	No	197 (45.4%)	39 (9.0%)	150 (46.2%)	29 (8.9%)	47 (43.1%)	10 (9.2%)
		Yes	19 (4.4%)	179 (41.2%)	16 (4.9%)	130 (40.0%)	3 (2.8%)	49 (45.0%)
		*p*-Value	0.013		0.074		0.092 †	

†—*p*-Value computed using exact binomial method.
